# Understanding Food Consumption Frequencies Among U.S. Chinese Older Adults

**DOI:** 10.1093/geroni/igaa057.780

**Published:** 2020-12-16

**Authors:** Shenglin Zheng, Dexia Kong, Qun Le, XinQi Dong

**Affiliations:** 1 Rutgers University, Chicago, Illinois, United States; 2 Rutgers University, New Brunswick, New Jersey, United States; 3 Rutgers University, New Brunswick,, New Jersey, United States

## Abstract

A healthy diet is essential to various health outcomes that are common among minority aging populations. To explore frequencies and correlates of food consumption among U.S. Chinese older adults, this study used data from the Population-based Study of Chinese Elderly (PINE) collected in Chicago during 2015-2017 (N=3053). Food consumption frequencies of five food groups (vegetables, fruits, grains, protein foods, and dairy) were assessed by a validated 48-item food frequency questionnaire. All responses of consumption frequency were transformed to “times per day” and weighted by reported portion size. The average frequencies of vegetables, grains, and protein foods intake among the U.S. Chinese older adults were 2.02 (SD±1.32), 1.32 (SD±0.70), and 1.58 (SD±0.90) times/day, respectively. Fruits and dairy consumption frequencies were much lower: 0.76 (SD±0.70) and 0.48 (SD±0.53) times/day. In addition, higher levels of education were correlated with higher consumption of all five food groups. Being female was positively correlated with frequencies of fruits and dairy intake. In contrast, poorer life quality and having more children were correlated with less intake of all five food groups. Older age, preference to speak Cantonese/Taishanese compared to Mandarin/English, and poorer health status were also correlated with lower consumption frequencies of fruit and dairy. The study provides important dietary data of U.S. Chinese older adults and sheds light on significant socioeconomic correlates of food consumption. More in-depth investigations are needed to clarify the sociocultural determinants of dietary behavior and how they relate to different health outcomes among the U.S. Chinese population.

